# Rapid detection of *Mycobacterium tuberculosis* using recombinase polymerase amplification: A pilot study

**DOI:** 10.1371/journal.pone.0295610

**Published:** 2023-12-08

**Authors:** Michael Sciaudone, Renzo Carpena, Maritza Calderón, Patricia Sheen, Mirko Zimic, Jorge Coronel, Robert H. Gilman, Natalie M. Bowman

**Affiliations:** 1 Department of Medicine, Section of Infectious Diseases, Tulane University School of Medicine, New Orleans, Louisiana, United States of America; 2 Center for Intelligent Molecular Diagnostics, Tulane University School of Medicine, New Orleans, Louisiana, United States of America; 3 Laboratorios de Investigación y Desarrollo, Facultad de Ciencias y Filosofía, Universidad Peruana Cayetano Heredia, Lima, Perú; 4 Department of International Health, Johns Hopkins University Bloomberg School of Public Health, Baltimore, Maryland, United States of America; 5 Division of Infectious Diseases, Department of Medicine, University of North Carolina at Chapel Hill School of Medicine, Chapel Hill, North Carolina, United States of America; Hangzhou Red Cross Hospital, CHINA

## Abstract

Tuberculosis remains one of the leading causes of death worldwide, especially in low- and middle-income countries. Tuberculosis treatment and control efforts are hindered by the difficulty in making the diagnosis, as currently available diagnostic tests are too slow, too expensive, or not sufficiently sensitive. Recombinase polymerase amplification (RPA) is a novel technique that allows for the amplification of DNA rapidly, at constant temperature, and with minimal expense. We calculated and compared the limit of detection, sensitivity, and specificity of two RPA-based assays for the diagnosis of pulmonary tuberculosis, using two sets of published primers. We also calculated and compared the assays’ limits of detection and compared their performance using two different DNA extraction methods prior to amplification (a commercially available DNA extraction kit vs. the chelex method). The RPA-lateral flow assay had a limit of detection of 5 fg/μL of DNA, a sensitivity of 53.2%, and a specificity of 93.3%, while the real time-RPA assay had a limit of detection of 25 fg/μL of DNA, a sensitivity of 85.1%, and a specificity of 93.3%. There was no difference in assay performance when DNA extraction was carried out using the commercial kit vs. the chelex method. The real-time RPA assay has adequate sensitivity and specificity for the diagnosis of pulmonary tuberculosis and could be a viable diagnostic tool in resource-limited settings, but the lateral flow assay did not perform as well, perhaps due to the fact we used stored sputum specimens from a biorepository. More work is needed to optimize the RPA-lateral flow assay, to get a more accurate estimate of its specificity and sensitivity using prospectively collected specimens, and to develop both assays into point-of-care tests that can be easily deployed in the field.

## Introduction

Tuberculosis (TB) remains one of the leading causes of death in the world and is the second leading cause of death from a single infectious agent, killing about 1.5 million people per year worldwide [1]. Unfortunately, establishing a diagnosis of TB remains challenging. Current diagnostic techniques include acid-fast smear, which lacks sensitivity and is highly operator-dependent, and culture on solid or liquid media, which takes weeks to result [2]. Polymerase chain reaction (PCR)-based assays, such as the Cepheid Xpert MTB/RIF and Ultra assays (Cepheid, Sunnyvale, CA, USA), and the Hain GenoType MTBDRplus and MTBDRsl line probe assays (Hain Lifescience GmbH, Nehren, Germany) have adequate sensitivity and specificity, yield more rapid results, and provide antimicrobial susceptibility data as well. Unfortunately, they rely on a stable power supply and regular maintenance and require expensive proprietary thermocyclers and cartridges, which makes them unsuitable for use as point-of-care (POC) or near POC assays in resource-limited settings, where the burden of TB tends to be the highest [3–7]. Additionally, TB diagnostics may perform differently in people living with HIV (PLWH) compared to HIV-negative individuals. Both sputum smear microscopy and Xpert MTB/RIF have lower sensitivity in this population [2, 8]. Detection of the *Mycobacterium tuberculosis* (MTB) cell wall antigen lipoarabinomannan (LAM) in urine is more sensitive in PLWH, but even so, its sensitivity is only 37%-61%, and it is sufficiently reliable only in people with very low CD4 cell counts [9–11].

TB diagnostic assays that have adequate sensitivity and specificity and are also cheap, rapid, and easy to perform are urgently needed. Isothermal nucleic acid amplification techniques such as loop-mediated amplification (LAMP), helicase-dependent amplification (HDA), and recombinase polymerase amplification (RPA) have emerged as attractive alternatives to PCR for use in resource-limited settings, due to their low cost, rapidity, and adaptability to POC assays [5, 6, 12–15]. RPA uses *Escherichia coli* recombinase, strand displacing DNA polymerase, and a single-stranded DNA-binding (SSB) protein, thus it does not depend on the thermal denaturation of a template and operates at a low and constant temperature, usually 25-42° C [3, 16]. In contrast, LAMP and HDA require a much higher amplification temperature (usually around 60–70°C) and demand a much stricter primer design [6, 14].

RPA has already been successfully used to amplify MTB DNA in the laboratory setting using serial dilutions of genomic DNA, as well as sputum samples spiked with genomic DNA [6, 17–22]. In this study, we used previously published RPA primers targeting IS1081 and IS6110 [17, 19], two highly conserved, repeated motifs unique to the MTB genome, to carry out RPA reactions and directly compare their limit of detection. We also applied the assay to remnant clinical sputum samples to compare results using two different nucleic acid extraction techniques prior to performing RPA. Then we obtained preliminary estimates of RPA’s sensitivity and specificity for MTB and directly compared the sensitivity and specificity of the RPA-LF and RT-RPA assays using sputum samples from a biorepository. The overarching goal was to obtain preliminary data that would inform further studies, with the aim of developing an RPA-based assay for TB with the highest possible sensitivity, specificity, and ease of use, and the lowest possible expense.

## Materials and methods

### Assay limit of detection

We determined the limit of detection of an RPA-lateral flow assay (RPA-LF) and real-time RPA (RT-RPA) using previously published primers and probes. The RPA-LF assay takes advantage of Endonuclease IV (nfo) and requires a target-specific probe labeled with fluorescein isothiocyanate (FITC) and a biotin-labeled reverse primer to enable detection of the amplification products with lateral flow technology. The RT-RPA assay uses an exonuclease and a probe labeled with a fluorophore and a quencher to generate a fluorescent signal in the presence of amplification. The fluorescent signal can be measured at regular intervals to generate amplification curves. Oligonucleotide primers and probes were purchased from Integrated DNA Technologies (IDT, Coralville, IA, USA). For the RPA-LF assay, we used the primers and probe published by Ma *et al*, which target IS1081 [19], while for the RT-RPA assay, we used two sets of primers and probes published by Boyle *et al*, which target IS1081 and IS6110 respectively (S1 Table) [17]. RPA reactions were carried out according to the manufacturer’s instructions using the TwistAmp nfo® and TwistAmp exo® kits (TwistDx Ltd., Cambridge, UK), for the RPA-LF and RT-RPA assays, respectively, with the following modifications: each reaction contained half of a lyophilized pellet, 14.75 μL of rehydration buffer, 4.1 μL of molecular biology grade water, 1.05 μL of forward primer (10 μM), 1.05 μL of reverse primer (10 μM), 0.3 μL of probe (10 μM), 2.5 μL of genomic DNA from MTB strain CDC1551 (BEI resources, NIAID, NIH) in serial dilutions, and 1.25 μL of 280 mM magnesium acetate, for a total reaction volume of 25 μL. Molecular biology grade water (2.5 μL) was used as a negative control instead of DNA. For the RPA-LF assay, the reactions were incubated on a heating block at 37° C for 30 minutes, after which 10 μL of the reaction products were diluted in 140 μL of PCRD buffer and a PCRD lateral flow strip (Abingdon Health, York, UK) was dipped into the solution. For the RT-RPA assay, reactions were incubated at 37°C for 30 minutes using a LightCycler® 96 (Roche Diagnostics Corporation, Indianapolis, IN, USA) combined heating and fluorescence detection device, with fluorescence measurements taken every minute.

### Sputum decontamination and DNA extraction

To determine the ideal nucleic acid extraction method, we compared the performance of RPA after extracting DNA using the chelex method or a commercially available extraction kit using 19 de-identified sputum samples (of which 9 were positive by both auramine stain and MODS, and 10 were negative by both methods). These were discarded remnants of specimens collected for routine diagnostic testing in clinics throughout Lima, Peru, and contained no information that would enable identification of the source patients. First, the sputa were decontaminated using the NALC-NaOH method [23]. Microscopic Observation Drug Susceptibility (MODS) culture [24] and auramine stain were performed. DNA was then extracted from the remnant of each decontaminated sample using the Roche High Pure PCR Template Preparation Kit (Roche Diagnostics Corporation, Indianapolis, IN, USA) according to the manufacturer’s instructions, and/or the chelex method. Briefly, for the chelex method, 300 μL of decontaminated sputum sample were centrifuged at 12,000 rpm for 10 minutes. The supernatant was discarded, and the sample was left to dry for 15 minutes. The sample was then resuspended in 80 μL of Chelex-10% Triton-X 100–1% TE buffer, vortexed, incubated at 100° C for 20 minutes, then brought to -20°C for 10 minutes. Finally, the sample was centrifuged at 7,000 rpm for 5 minutes. The supernatant was then used for RPA reactions, which were carried out as described above. Sensitivity and specificity were calculated by constructing contingency tables, using a composite of MODS and microscopy as the reference standard. We chose this to maximize the sensitivity of the reference standard, as currently there is no perfect reference standard for TB, and to approximate as closely as possible how the assay would perform in a clinical scenario, since, in most cases, either a positive culture or positive microscopy would be enough to trigger a decision to start antituberculous treatment.

### Sensitivity and specificity of RPA

We used 62 de-identified sputum samples from a biorepository. These specimens were collected from patients undergoing evaluation for suspected TB at outpatient clinics in Lima, Peru, between August and September 2020 and were originally used in a study aimed at evaluating the sensitivity and specificity of MODS culture at neutral pH to detect pyrazinamide resistance [25]. The participants had provided written consent for storage of these samples and for their use in future studies. Informed consent was not obtained for this study, as the samples were de-identified, which made it impossible to contact participants to obtain further consent. These samples had already been decontaminated prior to storage using the NALC-NaOH method and been analyzed by smear microscopy and MODS. RPA reactions were carried out in January 2023. Those who performed the RPA assays on these samples were unaware of smear microscopy and MODS results. First, we extracted DNA using the chelex method as described above. The amount and purity of the extracted DNA were measured using a NanoDrop 2000 spectrophotometer (Thermo Fisher Scientific). Extracted DNA was stored at -20° C immediately after extraction and thawed immediately prior to being used for RPA reactions. All RPA reactions were carried out between 24 and 72 hours after DNA extraction. RPA-LF reactions were carried out described as above, using 2.5 μL of DNA extracted from sputum samples instead of genomic DNA, and using the primers and probe published by Ma *et al*, [19] targeting IS1081. RT-RPA reactions were also carried out as above, using the primers and probe published by Boyle *et al*, targeting IS6110 [17]. Sensitivity and specificity were calculated by constructing contingency tables, using a composite of MODS and microscopy as the reference standard. For the RT-RPA assay, we also used ordinal logistic regression to assess for associations between the intensity of the fluorescent signal measured by the fluorometer and the number of days it took before growth was detected on MODS, or the mycobacterial load observed on microscopy (negative, 1+, 2+, or 3+).

## Results

### Assay limit of detection

**Fig 1:**
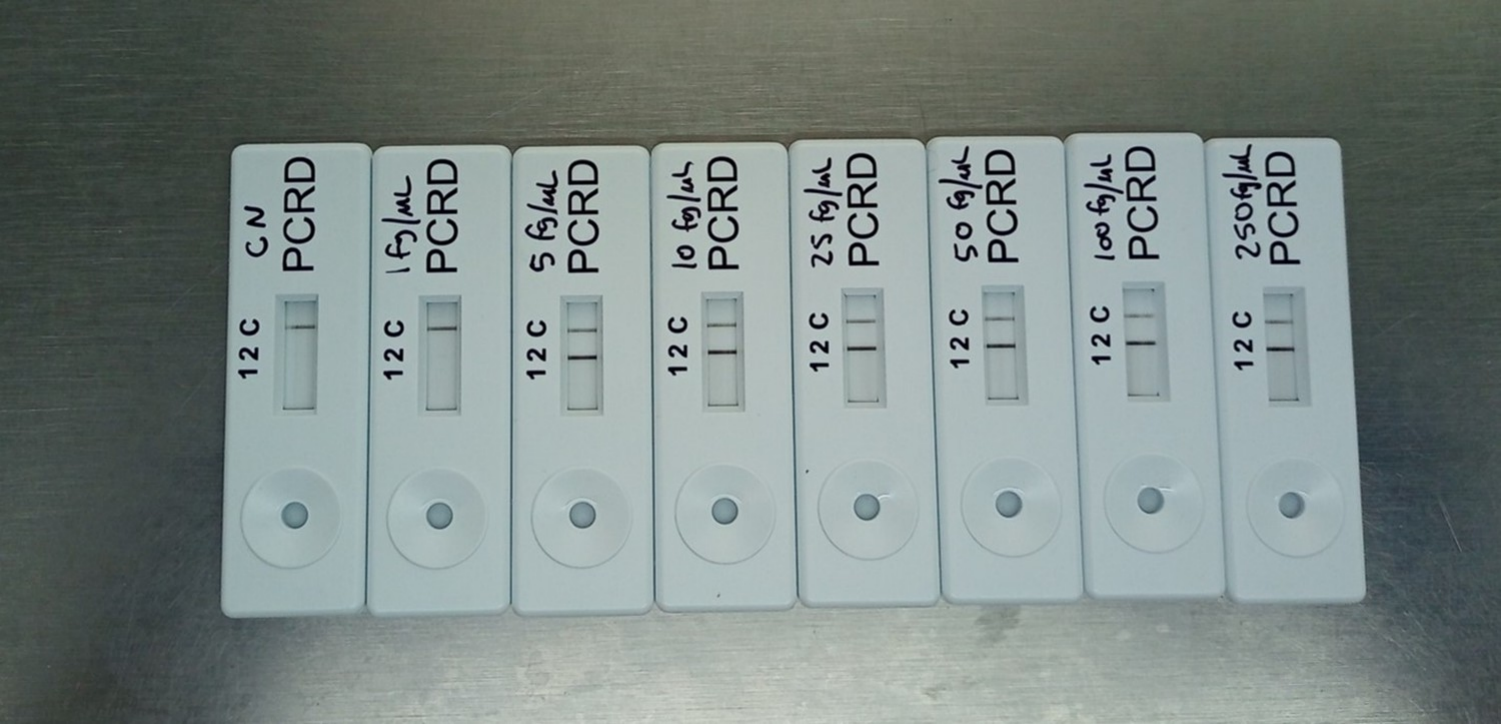
Limit of detection of the RPA-LF assay. Negative control (labeled CN) is shown on the left, and serial dilutions of MTB genomic DNA are shown from left to right in the following order: 1 fg/μL, 5 fg/μL, 10 fg/μL, 25 fg/μL, 50 fg/μL, 100 fg/μL, 250 fg/μL. The appearance of a control and a test line indicates a positive result (5 fg/μL to 250 fg/μL), while the appearance of the control line alone (negative control and 1 fg/μL) signifies no amplification occurred.

**Fig 2:**
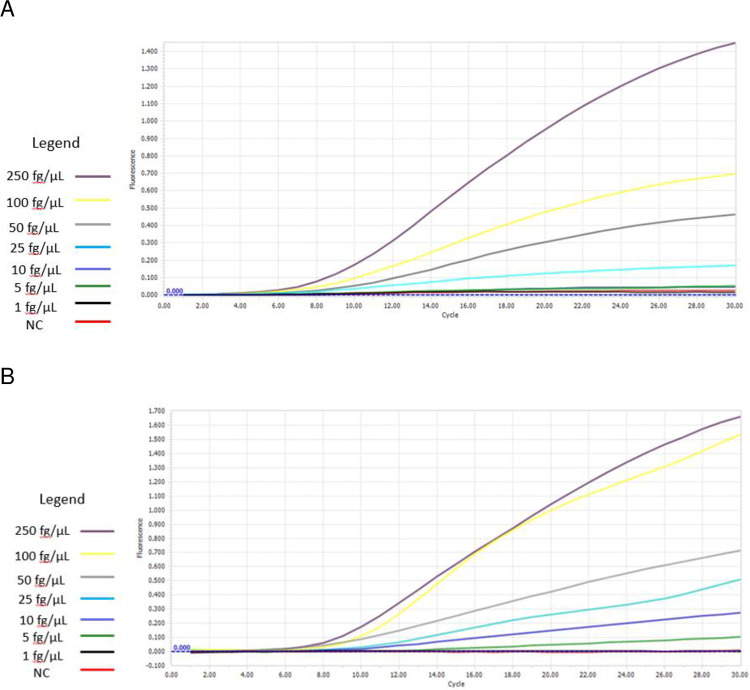
Representative amplification curves showing real-time amplification of IS1081 (panel A) and IS6110 (panel B), using serial dilutions of MTB genomic DNA.

RPA-LF reactions were carried out using serial dilutions of MTB genomic DNA from 1 fg/μL to 250 fg/μL, and molecular biology grade water as the negative control. Each reaction was carried out in triplicate and yielded the same results each time. The lowest limit of detection of the RPA-LF assay using primers targeting IS1081 was 5 fg/μL of DNA, which is equivalent to approximately one MTB genome/μL. An example of the results is shown in Fig 1. RT-RPA assays using primers targeting IS6110 and IS1081 were carried out in triplicate. Results are summarized in Table 1. When using the IS1081 primers, the lowest concentration of DNA that consistently yielded positive results was 50 fg/μL, but concentrations as low as 25 fg/μL were detected on 2 out of 3 repetitions. Using the IS6110 primers, consistently positive results were obtained at DNA concentrations ≥25 fg/μL, and sometimes at concentrations as low as 5 fg/μL. As expected, the intensity of the fluorescence generated correlated positively with the sample DNA concentration, and the time to onset of amplification increases as the DNA concentration decreases (Fig 2).

**Table 1:** Limit of detection of the RT-RPA assays using serial dilutions of MTB strain CDC 1551 genomic DNA and primers/probes targeting IS6110 and IS1081. The number of positive results obtained out of 3 repetitions is shown. NC = negative control.

	DNA Concentration								
Target Sequence		NC	1 fg/μL	5 fg/μL	10 fg/μL	25 fg/μL	50 fg/μL	100 fg/μL	250 fg/μL
	IS6110	0/3	0/3	1/3	1/3	3/3	3/3	3/3	3/3
	IS1081	0/3	0/3	0/3	0/3	2/3	3/3	3/3	3/3

### Comparison of extraction methods

DNA was extracted from 16 sputum samples using the Roche High Pure PCR Template Preparation Kit, and from 14 samples using the chelex method. Results of the MODS, auramine stain, and RPA are shown in Table 2. After DNA extraction with the chelex method, RPA yielded one false negative result and the calculated sensitivity and specificity were 85.7% and 100% respectively (Table 2A). After DNA extraction with the commercial kit, RPA yielded one false positive and one false negative result. The calculated specificity and sensitivity were 83.3% and 90% respectively (Table 2B). MODS, auramine stain, and RPA results for each sample are shown in S2 Table. Of note, one sample was classified as paucibacillary on auramine stain and yielded a false negative RPA result after extraction with both the commercial kit and the chelex method. Since the results were comparable, we used the chelex method for further experiments.

**Table 2:** Contingency table comparing results of MODS (reference standard) and RPA-LF performed after DNA extraction with the chelex method (panel A) and with the commercial kit (panel B).

A			
	TB(+)	TB (-)	Total
RPA-LF positive	6	0	6
RPA-LF negative	1	7	8
Total	7	7	14
B			
	TB (+)	TB (-)	Total
RPA-LF positive	5	1	6
RPA-LF negative	1	9	10
Total	6	10	16

Sensitivity = 85.7%, specificity = 100%

Sensitivity = 83.3%, specificity = 90%

### Sensitivity and specificity of RPA

We extracted DNA and conducted RPA reactions on 62 sputum samples. The amount of DNA extracted from each sample is shown in S3 Table. Forty-seven samples were positive by either MODS or microscopy. Two were smear-negative, 15 were 1+ on smear microscopy, 14 were 2+, and 16 were 3+. Forty were positive by MODS, of which 19were positive at 7 days, 11 were positive at 9 days, and 10 were positive after 10 or more days of incubation. The results of the RPA-LF assay and RT-RPA assay are summarized in Table 3. The RPA-LF assay had a sensitivity of 53.2% (95% CI 40.8%-65.6%) and a specificity of 93.3% (95% CI 87.1%-99.5%), while the RT-RPA assay had a sensitivity of 85.1% (95% CI 76.2%-94.0%) and a specificity of 93.3% (95% CI 87.1%-99.5%). Results for each individual sample are shown in S4 Table. We also calculated the assays’ sensitivities according to microscopy results and to the number of days it took before growth was detected on MODS to verify whether sensitivity was correlated with mycobacterial load. The sensitivity of the RPA-LF assay was 40.0%, 71.4%, and 50.0% for 1+, 2+, and 3+ smears, respectively. The sensitivity of the RT-RPA assay was 86.7%, 78.6% and 93.8% for 1+, 2+, and 3+ smears, respectively (S5 Table). Among the MODS-positive samples, the sensitivity of the RPA-LF assay was 57.9% for samples that were positive at 7 days, 45.5% for samples that were positive at 9 days, and 50.0% for samples that were positive at 10 days or more. The RT-RPA assay had a sensitivity of 94.7%, 90.9%, and 70.0% among samples that were positive at 7, 9, and 10 or more days, respectively (S6 Table). In the RT-RPA assay, higher mycobacterial load and shorter time to MODS positivity were associated with more intense fluorescence (p < 0.01 and p = 0.03 respectively).

**Table 3:** Contingency table comparing results of MODS (reference standard) and RPA-LF assay (panel A) and comparing results of MODS and RT-RPA (panel B).

A			
	TB(+)	TB (-)	TOTAL
RPA-LF (+)	25	1	26
RPA-LF (-)	22	14	36
TOTAL	47	15	62
B			
	TB (+)	TB (-)	TOTAL
RT- RPA (+)	40	1	41
RT-RPA (-)	7	14	21
TOTAL	47	15	62

Sensitivity = 53.2% (95% CI 40.8%-65.6%), specificity = 93.3% (95%

CI 87.1%-99.5%)

Sensitivity = 85.1% (95% CI 76.2%-94.0%), specificity = 93.3% (95%

CI 87.1%-99.5%)

## Discussion

We established and directly compared the lowest limit of detection of RPA-LF and RT-RPA assays using three sets of previously published primers and probes directed at MTB. Furthermore, we compared two nucleic acid extraction methods prior to RPA, and found that they produce similar results in terms of sensitivity and specificity of the assay. Finally, we used stored sputum samples to obtain an estimate of the assay’s sensitivity and specificity for the diagnosis of pulmonary TB, and directly compared two sets of primers/probes using two amplification detection platforms.

Our findings provide further corroboration that RPA is a promising platform to detect low concentrations of MTB DNA, which makes it an attractive alternative to currently available TB diagnostics, especially in resource-limited settings where rapid, cheap, easy-to-perform, and sensitive assays are urgently needed. Of note, the RPA-LF assay had a lower limit of detection than the RT-RPA assay for MTB. This confirms previously published data [17], and is likely explained by the fact that the exonuclease present in the RT-RPA assay somewhat lowers the reaction’s efficiency.

Initially, we extracted DNA from a small subset of sputum samples using two different methods and found that the RPA-LF assay had adequate sensitivity and specificity (>80% and >90% respectively) for the detection of MTB from sputum, regardless of the nucleic acid extraction method used. Thus, it seems that using an easier and less expensive nucleic acid extraction method is not detrimental to the subsequent RPA assay’s sensitivity, compared to extraction performed with a commercially available kit. This seems promising, as it allows for minimization of the assay’s total cost without sacrificing sensitivity.

When we performed the assays on a larger number of stored sputum samples, however, the RPA-LF assay performed poorly compared to the RT-RPA assay, which was an unexpected result. We speculate that this may be explained by the fact that some of the samples in the larger group may have been stored for longer and DNA may have partially degraded, or the initial results may not be accurate due to small sample size. More importantly, interpretation of lateral flow results is more subjective, as positive samples with small amounts of DNA can produce very faint test lines, which may be misinterpreted as a negative result, despite all the measures taken to minimize this possibility. The RT-RPA assay on the other hand, is less susceptible to this type of bias, as the fluorescent signal is measured by a fluorometer and the cutoff value for positivity is determined a priori. Another possible explanation is that the RPA-LF assay involves more steps in which the tubes are opened and the sample is pipetted and transferred, thus increasing the chances for contamination and introduction of inhibitors.

Despite these limitations, the RPA-LF assay provides an easier modality for result read-out and thus has greater potential to be adapted into a POC or near-POC assay, since, unlike the RT-RPA assay, it does not require a fluorometer. Further investigation is needed to increase the assay’s sensitivity, perhaps by finding more sensitive primers, by using a CRISPR-Cas system to increase amplicon detection [26], or by creating a POC assay platform which minimizes tube opening and pipetting steps, similar to Mondal *et al*’s mobile laboratory [27]. On the other hand, RT-RPA has some advantages, namely, the ability to generate amplification curves, and it could be developed into a quantitative or semi-quantitative assay to estimate the burden of disease or monitor response to treatment. In fact, while the sensitivity of both the RPA-LF and RT-RPA assays was not proportional to mycobacterial load as measured by smear microscopy or by time to MODS positivity, the strength of the fluorescent signal generated in the RT-RPA assay was inversely proportional to time to MODS positivity and directly proportional to the mycobacterial load observed on microscopy, as was expected. This provides stronger support to the viability of RPA as a diagnostic test for TB and it portends the possibility of using RT-RPA as a quantitative assay. The failure to detect differences in sensitivity based on mycobacterial load and time to MODS positivity may have been due to insufficient power to compare smaller groups of samples. A larger study using freshly collected samples from patients undergoing diagnostic evaluation for TB is needed to calculate both assays’ operating characteristics more accurately and conduct sub-group analyses.

Of note, we had one sample that was classified as paucibacillary on auramine stain and yielded a false negative RPA result regardless of the DNA extraction method used (S2 Table). In the larger group, we had two smear-negative but culture-positive samples, one of which was positive by both RT-RPA and RPA-LF, while the other yielded a false negative result in both assays (S4 Table). More paucibacillary specimens, as well as smear-negative/culture-positive ones, will need to be tested to ascertain RPA’s sensitivity in patients with low mycobacterial loads. This is particularly important, because the ability to detect smear-negative TB cases is what would make RPA a formidable tool to diagnose a greater number of patients earlier and start them on the correct therapy, especially in resource-limited settings.

While this is only a pilot study, limited by its small sample size, it serves as proof of concept that RPA is a viable platform to detect MTB in clinical samples, and it provides useful preliminary data which could serve as the basis for further development of an RPA assay for TB. A study using a larger number of prospectively collected sputum samples is needed to better ascertain the assay’s operating characteristics in a clinical scenario. Furthermore, the assay’s sensitivity could be increased by pairing it with CRISPR-based amplicon detection, which has yielded very promising results with other pathogens [26]. While this would make the assay more complex and would somewhat increase its cost, it may yield a sensitivity comparable to that of PCR and may allow for the use of RPA for the diagnosis of extra-pulmonary TB syndromes in which mycobacterial loads are typically lower, such as meningitis. In fact, the development of rapid point-of-care diagnostic tests for extra-pulmonary TB has been identified by the World Health Organization (WHO) as a critical research priority [11].

Furthermore, RPA could be adapted into an assay for multi-drug resistant TB (MDR-TB) by using allele-specific primers to selectively amplify genes carrying antimicrobial resistance-conferring mutations. This possibility has been demonstrated in the laboratory setting with promising results [3, 6, 20], but larger studies using clinical samples are needed to establish whether RPA would be a suitable platform to accurately and rapidly identify MDR-TB. Perhaps the most important issue that remains to be addressed to make RPA a truly useful diagnostic tool is the adaptation of the assay for use in the field or in very resource-limited settings. RPA is fairly easy to perform and requires minimal training. Based on our experience, a lone technician would be able to complete all the necessary steps (DNA extraction, RPA reaction preparation, incubation, and read-out) in under 2 hours, using only a hood for DNA extraction and reaction preparation and a simple heating block for incubation (as well as a fluorometer for the RT-RPA assay). However, further adaptation is needed to turn RPA into a truly point-of-care assay.

In summary, RPA can detect low concentrations of MTB DNA under ideal laboratory settings and seems to have adequate sensitivity and specificity for the detection of pulmonary TB based on a small number of clinical sputum samples, but larger, prospective studies are needed to better evaluate its operating characteristics for the diagnosis of both pulmonary and extra-pulmonary TB, and the assay requires further adaptation for use as a POC test.

## Supporting information

S1 Table RPA primers and probes sequences. FITC = fluorescein isothiocyanate, H = tetrahydrofuran spacer, P = 3’ phosphate to block elongation, F = dT-FAM, H = tetra hydrofuran and Q = dT-Black Hole Quencher 1.
(DOCX)

S2 Table Comparison of the results of MODS, auramine stain, and RPA performed after DNA extraction with the chelex method and the commercial extraction kit. False positive and false negative results are bolded. NP = not performed due to insufficient sample quantity.
(DOCX)

S3 Table Quantification of total DNA extracted from each sample. (DOCX)

S4 Table Results of MODS, smear microscopy, RPA-LF, and RT-RPA of each individual sample. False positive and false negative results are bolded.
(DOCX)

S5 Table Sensitivity of the RT-RPA assay (panel A) and RPA-LF assay (panel B) according to smear microscopy result.
(DOCX)

S6 Table Sensitivity of the RT-RPA assay (panel A) and RPA-LF assay (panel B) according to time (in days) to MODS positivity.
(DOCX)
