# Supplementary material for: Rapid detection of Mycobacterium tuberculosis using recombinase polymerase amplification: A pilot study
Source: PLoS One. 2023 Dec 8;18(12):e0295610. doi: 10.1371/journal.pone.0295610 (PMC10707601; doi:10.1371/journal.pone.0295610)
Supplement: S1 Table — FITC = fluorescein isothiocyanate, H = tetrahydrofuran spacer, P = 3’ phosphate to block elongation, F = dT-FAM, H = tetra hydrofuran and Q = dT-Black Hole Quencher 1. (DOCX) [file pone.0295610.s001.docx]

| RPA-LF^22^ |  | Target Sequence | Primer/Probe Sequence (5’ to 3’) |
| --- | --- | --- | --- |
|  | Forward Primer | IS1081 | CCAAGCTGCGCCAGGGCAGCTATTTCCCGGAC |
|  | Reverse Primer | IS1081 | Biotin-TTGGCCATGATCGACACTTGCGACTTGGA |
|  | Probe | IS1081 | FITC-GAACGCGCACTGACCAGCGTGGTGGCGACCTG(H)TACCTGCTGGGAGTATC-P |
| RT-RPA^20^ |  |  |  |
|  | Forward Primer | IS1081 | CAGTAGTGGGCGGTCATCGCGTGATCCTTCGAAACGACC |
|  | Reverse Primer | IS1081 | CTCGCCTGTGCGAGTTGGTCAGCCAGAAGCTG |
|  | Probe | IS1081 | CGATAAGATGAGAAGAGGTCATTGCGTCATT(F)(H)C(Q)TCGATTGACTTTTGCT-Spacer C3 |
|  | Forward Primer | IS6110 | GATCCTGCGAGCGTAGGCGTCGGTGACAAAGGCCACGTAG |
|  | Reverse Primer | IS6110 | CTGATCCGGCCACAGCCCGTCCCGCCGATCTCGTCCAGC |
|  | Probe | IS6110 | CGAACCCTGCCCAGGTCGACACATAGGTGAGGTC(F)(H)C(Q)ACCCACAGCCGGTTA-Spacer C3 |
