# Supplementary material for: Rapid detection of Mycobacterium tuberculosis using recombinase polymerase amplification: A pilot study
Source: PLoS One. 2023 Dec 8;18(12):e0295610. doi: 10.1371/journal.pone.0295610 (PMC10707601; doi:10.1371/journal.pone.0295610)
Supplement: S2 Table — False positive and false negative results are bolded. NP = not performed due to insufficient sample quantity. (DOCX) [file pone.0295610.s002.docx]

| Sample Number | MODS Result | Auramine Stain Result | RPA Result after DNA extraction with the chelex method | RPA Result after DNA extraction with a commercial kit |
| --- | --- | --- | --- | --- |
| 1 | Positive | 2+ | Positive | Positive |
| 2 | Positive | 3+ | Positive | Positive |
| 3 | Positive | 2+ | Positive | Positive |
| 4 | Positive | Paucibacillary | **Negative** | **Negative** |
| 5 | Negative | Negative | Negative | Negative |
| 6 | Negative | Negative | Negative | Negative |
| 7 | Positive | 2+ | Positive | NP |
| 8 | Positive | 2+ | Positive | NP |
| 9 | Positive | 2+ | Positive | NP |
| 10 | Negative | Negative | Negative | Negative |
| 11 | Negative | Negative | Negative | Negative |
| 12 | Negative | Negative | Negative | Negative |
| 13 | Negative | Negative | Negative | Negative |
| 14 | Negative | Negative | Negative | **Positive** |
| 15 | Negative | Negative | Negative | Negative |
| 16 | Positive | 3+ | NP | Positive |
| 17 | Positive | 3+ | NP | Positive |
| 18 | Negative | Negative | NP | Negative |
| 19 | Negative | Negative | NP | Negative |
