# Supplementary material for: Rapid detection of Mycobacterium tuberculosis using recombinase polymerase amplification: A pilot study
Source: PLoS One. 2023 Dec 8;18(12):e0295610. doi: 10.1371/journal.pone.0295610 (PMC10707601; doi:10.1371/journal.pone.0295610)
Supplement: S3 Table — (DOCX) [file pone.0295610.s003.docx]

| Sample number | DNA (ng/µL) |
| --- | --- |
| 1 | 152.4 |
| 2 | 714.2 |
| 3 | 148.2 |
| 4 | 57.3 |
| 5 | 64.7 |
| 6 | 733.2 |
| 7 | 38.0 |
| 8 | 84.3 |
| 9 | 135.7 |
| 10 | 92.7 |
| 11 | 57.8 |
| 12 | 77.5 |
| 13 | 95.1 |
| 14 | 27.2 |
| 15 | 88.0 |
| 16 | 106.7 |
| 17 | 21.1 |
| 18 | 17.6 |
| 19 | 15.9 |
| 20 | 71.3 |
| 21 | 43.5 |
| 22 | 140.0 |
| 23 | 106.9 |
| 24 | 53.6 |
| 25 | 25.5 |
| 26 | 53.5 |
| 27 | 73.2 |
| 28 | 86.8 |
| 29 | 90.1 |
| 30 | 92.2 |
| 31 | 85.5 |
| 32 | 57.1 |
| 33 | 169.5 |
| 34 | 52.2 |
| 35 | 47.6 |
| 36 | 51.1 |
| 37 | 5.2 |
| 38 | 18.4 |
| 39 | 6.7 |
| 40 | 214.5 |
| 41 | 218.1 |
| 42 | 0.0 |
| 43 | 90.5 |
| 44 | 24.2 |
| 45 | 37.3 |
| 46 | 22.1 |
| 47 | 107.8 |
| 48 | 380.4 |
| 49 | 123.6 |
| 50 | 31.8 |
| 51 | 188.2 |
| 52 | 140.1 |
| 53 | 71.8 |
| 54 | 203.6 |
| 55 | 35.9 |
| 56 | 92.6 |
| 57 | 288.1 |
| 58 | 136.0 |
| 59 | 94.2 |
| 60 | 354.8 |
| 61 | 30.3 |
| 62 | 87.2 |
