# Supplementary material for: Rapid detection of Mycobacterium tuberculosis using recombinase polymerase amplification: A pilot study
Source: PLoS One. 2023 Dec 8;18(12):e0295610. doi: 10.1371/journal.pone.0295610 (PMC10707601; doi:10.1371/journal.pone.0295610)
Supplement: S4 Table — False positive and false negative results are bolded. (DOCX) [file pone.0295610.s004.docx]

| Sample Number | MODS Result | Microscopy Result | RPA-LF Result | RT-RPA Result |
| --- | --- | --- | --- | --- |
| 1 | Positive | 3+ | **Negative** | Positive |
| 2 | Negative | Negative | Negative | Negative |
| 3 | Positive | 1+ | **Negative** | Positive |
| 4 | Positive | 3+ | Positive | Positive |
| 5 | Negative | 1+ | Positive | Positive |
| 6 | Negative | Negative | Negative | Negative |
| 7 | Positive | 2+ | Positive | Positive |
| 8 | Positive | 1+ | **Negative** | Positive |
| 9 | Positive | 2+ | Positive | Positive |
| 10 | Negative | Negative | Negative | **Positive** |
| 11 | Negative | Negative | Negative | Negative |
| 12 | Positive | 1+ | Positive | Positive |
| 13 | Positive | 1+ | **Negative** | Positive |
| 14 | Positive | 3+ | Positive | Positive |
| 15 | Positive | 1+ | **Negative** | Positive |
| 16 | Negative | Negative | Negative | Negative |
| 17 | Negative | 3+ | Positive | Positive |
| 18 | Positive | 3+ | Positive | Positive |
| 19 | Positive | 3+ | Positive | Positive |
| 20 | Positive | 3+ | Positive | Positive |
| 21 | Positive | 1+ | **Negative** | **Negative** |
| 22 | Negative | Negative | Negative | Negative |
| 23 | Negative | Negative | Negative | Negative |
| 24 | Positive | 1+ | Positive | Positive |
| 25 | Positive | 1+ | Positive | Positive |
| 26 | Positive | 2+ | Positive | Positive |
| 27 | Positive | 3+ | Positive | Positive |
| 28 | Negative | Negative | Negative | Negative |
| 29 | Negative | 1+ | **Negative** | Positive |
| 30 | Positive | 2+ | Positive | Positive |
| 31 | Positive | 1+ | **Negative** | Positive |
| 32 | Positive | 1+ | **Negative** | **Negative** |
| 33 | Negative | Negative | Negative | Negative |
| 34 | Positive | 3+ | **Negative** | Positive |
| 35 | Positive | 2+ | **Negative** | Positive |
| 36 | Positive | 1+ | Positive | Positive |
| 37 | Positive | 3+ | Positive | Positive |
| 38 | Negative | Negative | Negative | Negative |
| 39 | Positive | 2+ | Positive | Positive |
| 40 | Negative | 3+ | **Negative** | Positive |
| 41 | Positive | 3+ | **Negative** | Positive |
| 42 | Positive | 2+ | Positive | Positive |
| 43 | Positive | 2+ | Positive | Positive |
| 44 | Negative | Negative | **Positive** | Negative |
| 45 | Positive | **Negative** | **Negative** | **Negative** |
| 46 | Positive | 1+ | Positive | Positive |
| 47 | Positive | 2+ | Positive | Positive |
| 48 | Negative | Negative | Negative | Negative |
| 49 | Positive | 1+ | **Negative** | Positive |
| 50 | Positive | **Negative** | Positive | Positive |
| 51 | Negative | Negative | Negative | Negative |
| 52 | Contaminated | 2+ | **Negative** | **Negative** |
| 53 | Negative | Negative | Negative | Negative |
| 54 | Positive | 3+ | **Negative** | Positive |
| 55 | Positive | 2+ | Positive | **Negative** |
| 56 | Negative | 2+ | **Negative** | **Negative** |
| 57 | Positive | 2+ | **Negative** | Positive |
| 58 | Positive | 3+ | **Negative** | Positive |
| 59 | Positive | 3+ | **Negative** | **Negative** |
| 60 | Positive | 3+ | **Negative** | Positive |
| 61 | Negative | 2+ | Positive | Positive |
| 62 | Negative | Negative | Negative | Negative |
