# Supplementary material for: Rapid detection of Mycobacterium tuberculosis using recombinase polymerase amplification: A pilot study
Source: PLoS One. 2023 Dec 8;18(12):e0295610. doi: 10.1371/journal.pone.0295610 (PMC10707601; doi:10.1371/journal.pone.0295610)
Supplement: S5 Table — Sensitivity of the RT-RPA assay (panel A) and RPA-LF assay (panel B) according to smear microscopy result. (DOCX) [file pone.0295610.s005.docx]

A.

|  | 1+ smear | 2+ smear | 3+ smear | Total |
| --- | --- | --- | --- | --- |
| RPA (+) | 13 | 11 | 15 | 39 |
| RPA (-) | 2 | 3 | 1 | 6 |
| Total | 15 | 14 | 16 | 45 |
| Sensitivity | 86.7% (74.9%-  98.4% | 78.6% (64.1%-  93.0%) | 93.8% (85.5%-  100%) |  |

B.

|  | 1+ smear | 2+ smear | 3+ smear | Total |
| --- | --- | --- | --- | --- |
| RPA (+) | 6 | 10 | 8 | 24 |
| RPA (-) | 9 | 4 | 8 | 21 |
| Total | 15 | 14 | 16 | 45 |
| Sensitivity | 40.0% (23.0%-  57.0%) | 71.4% (55.5%-  87.3%) | 50.0% (32.9%-  67.0%) |  |
