# Supplementary material for: Rapid detection of Mycobacterium tuberculosis using recombinase polymerase amplification: A pilot study
Source: PLoS One. 2023 Dec 8;18(12):e0295610. doi: 10.1371/journal.pone.0295610 (PMC10707601; doi:10.1371/journal.pone.0295610)
Supplement: S6 Table — Sensitivity of the RT-RPA assay (panel A) and RPA-LF assay (panel B) according to time (in days) to MODS positivity. (DOCX) [file pone.0295610.s006.docx]

A.

|  | MODS (+) at 7 days | MODS (+) at 9 days | MODS (+) at 10 or more days | Total |
| --- | --- | --- | --- | --- |
| RPA (+) | 18 | 10 | 7 | 35 |
| RPA (-) | 1 | 1 | 3 | 5 |
| Total | 19 | 11 | 10 | 40 |
| Sensitivity | 94.7% (87.9%-  100% | 90.9% (81.1%-  100%) | 70.0% (54.1%-  85.9%) |  |

B.

|  | MODS (+) at 7 days | MODS (+) at 9 days | MODS (+) at 10 or more days | Total |
| --- | --- | --- | --- | --- |
| RPA (+) | 11 | 5 | 5 | 21 |
| RPA (-) | 8 | 6 | 5 | 19 |
| Total | 19 | 11 | 10 | 40 |
| Sensitivity | 57.9% (42.8%-  73.0%) | 45.5% (37.6%-  71.5%) | 50.0% (32.7%-  67.3%) |  |
